# Clinical and Genetic Characteristics of Finnish Patients with Autosomal Recessive and Dominant Non-Syndromic Hearing Loss Due to Pathogenic *TMC1* Variants

**DOI:** 10.3390/jcm11071837

**Published:** 2022-03-26

**Authors:** Minna Kraatari-Tiri, Maria K. Haanpää, Tytti Willberg, Pia Pohjola, Riikka Keski-Filppula, Outi Kuismin, Jukka S. Moilanen, Sanna Häkli, Elisa Rahikkala

**Affiliations:** 1 Department of Clinical Genetics, Oulu University Hospital, 90029 Oulu, Finland; minna.kraatari@oulu.fi (M.K.-T.); riikka.keski-filppula@ppshp.fi (R.K.-F.); outi.kuismin@ppshp.fi (O.K.); jukka.moilanen@oulu.fi (J.S.M.); 2 PEDEGO Research Unit, Medical Research Center Oulu, Oulu University Hospital, and University of Oulu, 90014 Oulu, Finland; sanna.hakli@ppshp.fi; 3 Department of Clinical Genetics, Turku University Hospital, 20521 Turku, Finland; maria.haanpaa@tyks.fi; 4 Department of Genomics, Turku University Hospital, 20521 Turku, Finland; pia.pohjola@tyks.fi; 5 Department of Otorhinolaryngology, Turku University Hospital, 20521 Turku, Finland; tytti.willberg@tyks.fi; 6 Department of Otorhinolaryngology, Oulu University Hospital, 90029 Oulu, Finland

**Keywords:** *TMC1*, hearing loss, cochlear implant, hearing rehabilitation, congenital

## Abstract

Sensorineural hearing loss (SNHL) is one of the most common sensory deficits worldwide, and genetic factors contribute to at least 50–60% of the congenital hearing loss cases. The transmembrane channel-like protein 1 (*TMC1*) gene has been linked to autosomal recessive (DFNB7/11) and autosomal dominant (DFNA36) non-syndromic hearing loss, and it is a relatively common genetic cause of SNHL. Here, we report eight Finnish families with 11 affected family members with either recessively inherited homozygous or compound heterozygous *TMC1* variants associated with congenital moderate-to-profound hearing loss, or a dominantly inherited heterozygous *TMC1* variant associated with postlingual progressive hearing loss. We show that the *TMC1* c.1534C>T, p.(Arg512*) variant is likely a founder variant that is enriched in the Finnish population. We describe a novel recessive disease-causing *TMC1* c.968A>G, p.(Tyr323Cys) variant. We also show that individuals in this cohort who were diagnosed early and received timely hearing rehabilitation with hearing aids and cochlear implants (CI) have reached good speech perception in noise. Comparison of the genetic data with the outcome of CI rehabilitation increases our understanding of the extent to which underlying pathogenic gene variants explain the differences in CI rehabilitation outcomes.

## 1. Introduction

Congenital moderate-to-profound bilateral sensorineural hearing loss (SNHL) affects at least 1 in 1000 infants [1], making SNHL is one of the most common sensory deficits worldwide. In developed countries, genetic hearing loss accounts for at least 50–60% of childhood hearing loss cases [2]. Inherited SNHL is often an isolated physical finding (non-syndromic SNHL) [3]. To date, more than 120 genes have been identified in association with monogenic non-syndromic hearing loss (https://hereditaryhearingloss.org/, accessed on 20 February 2022). In addition, hearing loss is associated with at least 400 different syndromes [4].

The transmembrane channel-like protein 1 (*TMC1*) gene encodes a transmembrane protein that acts as a component of the mechanotransduction channel in the auditory and vestibular hair cells of the inner ear [5]. *TMC1/2* mutant mice have no mechanosensitive current [6]. TMC1 is located at the tips of stereocilia of hair cells, where transduction occurs [7]. Pathogenic variants in *TMC1* alter the biophysical properties of hair cell mechanosensory transduction [8]. *TMC1* has been linked to autosomal recessive non-syndromic severe-to-profound hearing loss (DFNB7/11, OMIM #600974) and autosomal dominant late-onset progressive hearing loss (DFNA36, OMIM #606705) [9]. *TMC1* is a relatively common genetic cause of SNHL and accounts for 0.5–8.1% of patients with autosomal recessive SNHL (ARSNHL) in different ethnic populations [10,11,12].

In this study, we report eight Finnish families with 11 affected family members with recessively inherited homozygous, recessively inherited compound heterozygous, or dominantly inherited heterozygous *TMC1* variants.

## 2. Materials and Methods

### 2.1. Patient Recruitment

All individuals and their family members with pathogenic or likely pathogenic *TMC1* variants were contacted about the study, and all patients or their guardians gave their written informed consent for the research. The study was approved by the ethical review committee of Oulu University Hospital (EETTMK: 186/2020).

The patients were recruited from the departments of clinical genetics in Oulu University Hospital (Patients 1–8) and Turku University Hospital (Patients 9–11). In both clinics, genetic testing is offered to all children receiving a diagnosis of hearing loss, and to adults with hearing loss of unknown cause as a part of diagnostics.

### 2.2. Familial and Clinical Examination

The available medical records, as well as familial and clinical history, were evaluated. Eight (N = 8/11) affected individuals underwent an audiometry at 125–8000 Hz. Otoacoustic emission (OAE) testing (N = 6/11), transient evoked OAE (TEOAE) (N = 2/11), auditory brainstem response (ABR) (N = 6/11), and automated auditory brainstem response (AABR) (N = 2/11) were performed for the young individuals. These were not performed for the older individuals due to availability of the screening and different practices. Seven (N = 7/11) affected individuals underwent a speech-in-noise test. Speech perception was evaluated using spoken language; the adults conducted the Finnish matrix sentence test (FMST) and the children conducted the simplified Finnish matrix sentence test (FINSIMAT). Computed tomography (CT) scans and magnetic resonance imaging (MRI) were performed on those patients who had cochlear implants (CIs). The patients were subsequently referred to the Department of Clinical Genetics for pedigree construction and genetic evaluations.

### 2.3. Genetic Testing and Data Analysis

Peripheral blood samples were collected from available and consenting individuals, and genomic DNA was extracted using automated QIAsymphony device and Qiagen Qiasymphony DSP DNA Midi kit (Qiagen, Hilden, Germany). Traditionally, *GJB2* testing is performed before large gene panels. Six affected individuals were screened for pathogenic variants of *GJB2* by Sanger sequencing, with negative results.

Exome based hearing loss gene panels have been conducted over a period of last six years as part of clinical diagnostics. The gene panels performed vary depending on the laboratory where the test is conducted and the year the test is ordered. An exome-based next generation sequencing (NGS) hearing loss gene panels were performed in the Laboratory of Genome Diagnostics in Nijmegen, the Netherlands (gene panel versions DG-2.5 and DG-2.8) for two patients; Blueprint Genetics, Espoo, Finland (Comprehensive Hearing Loss and Deafness gene panel, version 6, 22 February 2020) for two patients; Tyks Genomics, Laboratory medicine, Turku, Finland for two patients, and Nordlab Oulu, Finland for two patients. In three families (Families 3, 5, and 8) with multiple participating affected individuals, targeted variant testing by Sanger sequencing was performed after the pathogenic variant was identified in the proband. In families with compound heterozygous *TMC1* variants, Sanger sequencing of parental samples was performed to confirm that the variants are in trans in patients. No other clinically relevant variants were found in the genetic testing.

## 3. Results

### 3.1. Clinical Characteristics of the Patients

**Figure 1:**
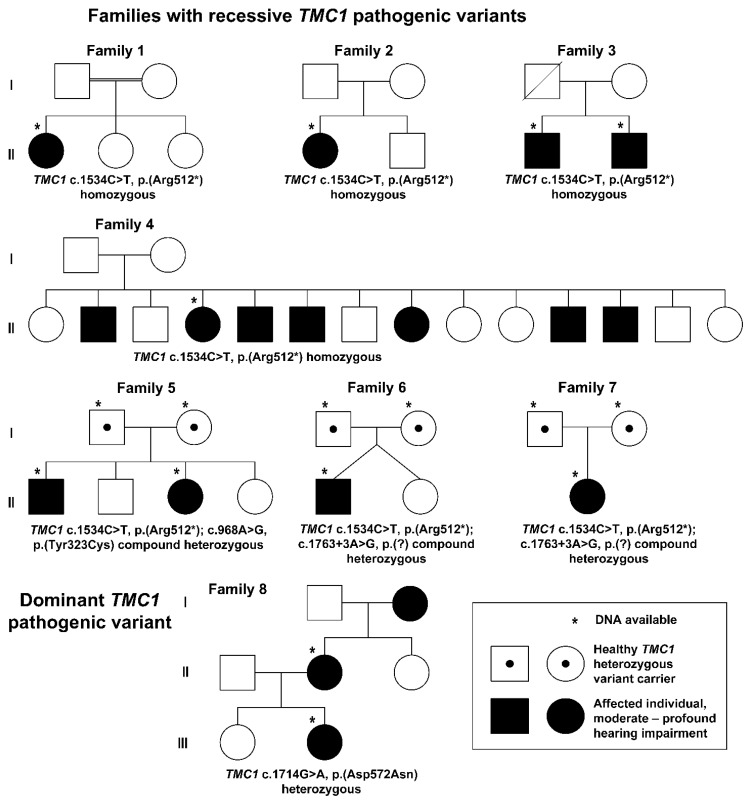
Pedigrees of the families in two (I–II) or three (I–III) generations. In six families, the *TMC1* c.1534C>T, p.(Arg512*); c.968A>G, p.(Tyr323Cys), and c.1763+3A>G, p.(?) variants segregated recessively. All the affected individuals showed profound congenital deafness. In one family, a dominantly inherited *TMC1* c.1714G>A, p.(Asp572Asn) segregated with the phenotype.

The clinical characteristics of the affected cases for each family included in this study are summarized in Table 1, and the pedigrees are shown in Figure 1. Eleven affected individuals from eight different Finnish families were recruited for the study. Four were males, and seven were females, and their ages ranged between 0 and 57 years.

**Figure 2:**
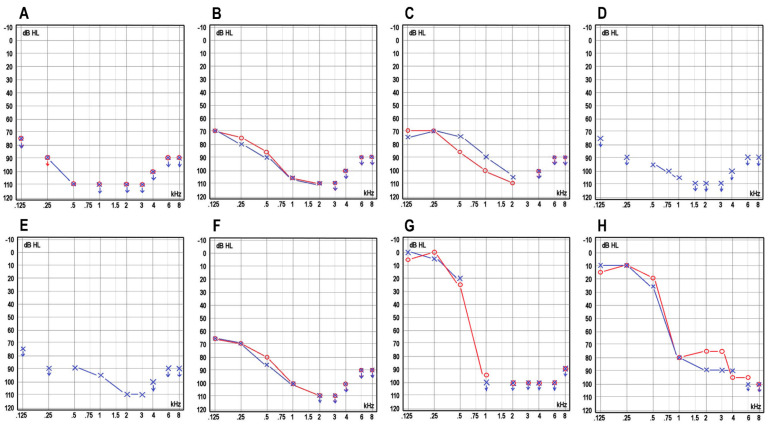
Pure tone audiograms (PTA) of the affected individuals. (**A**) Patient 1, (**B**) Patient 3, (**C**) Patient 5, (**D**) Patient 6, (**E**) Patient 7, and (**F**) Patient 8. All six of these patients showed severe-to-profound sensorineural hearing loss and mildly down-sloping curves associated with recessive pathogenic *TMC1* variants. (**G**) Patient 10 and (**H**) Patient 11 had deeply down-sloping curves, demonstrating predominant deterioration of higher frequencies associated with a dominant pathogenic *TMC1* variant. In all patients, the last available audiograms are shown. Patients 3, 5, and 11 have not received CIs. In Patients 1, 6, and 7, the audiograms shown are after the first, but before the second CI operation. In Patient 8 the audiogram shown is after bilateral CI operations. In Patient 10, the audiogram shown is before the CI operation. The red circles indicate the right ear and the blue X’s indicate the left ear.

In the families with a recessive pattern of inheritance (Families 1–7), the hearing loss was congenital. Three individuals (Patients 3–5 from Families 3 and 4) were born before the start of the newborn hearing screening program, and before CIs were available. Their hearing aid benefit was limited, and they all use sign language as their primary mode of communication. The newborn screenings were abnormal in all tested cases (N = 5/5, 100%), the OAE and TEOAE showed no responses (N = 6/6, 100% and (N = 2/2, 100%, respectively), and the ABR was abnormal in all tested cases (N = 6/6, 100%). An audiogram revealed a bilateral profound SNHL in all the affected individuals (Figure 2A–F). One individual reported a balance problem, but his motor development was normal. The ophthalmological examination revealed one individual with deuteranopia and one individual with esophoria and hyperopia. Imaging studies were unremarkable. Seven individuals were fitted with hearing aids (between 3 months to 1.5 years of age) but obtained limited benefits. Bilateral simultaneous or consecutive CIs were later implanted in five individuals at the ages of 12 months to 2 years, and bilateral simultaneous CIs are planned for the youngest individual at the age of 8–9 months. All implanted patients showed good progress in language acquisition. In the best-aided condition (bilateral CIs), in quiet, the speech recognition scores ranged from 75% to 100%. The two youngest patients, who were tested with the FINSIMAT, had speech reception thresholds (SRTs) of −6.6 dB signal-to-noise ratio (SNR) and −6.2 dB SNR. The best-aided SRTs for the adults, determined with the FMST, ranged from −3.4 dB SNR to −8.6 dB SNR.

In family 8, with dominant inheritance, the onset of hearing loss was postlingual and was diagnosed at the age of 7 years. The hearing loss was progressive, and audiograms revealed down-sloping curves (Figure 2G,H). The older affected individual benefited from CIs, which she received in her left ear at the age of 41 and in her right ear at the age of 51. Her best-aided SRT with FMST was −2.8 dB SNR. The younger affected individual has been offered CIs but currently prefers to use hearing aids. Her best-aided SRT with the FMST was +0.4 dB SNR.

### 3.2. Genetic Results

Five individuals from families 1 to 4 were homozygous for the *TMC1* (NM_138691.3) c.1534C>T, p.(Arg512*) (hg38: chr9:72792320C>T, rs200171616) variant, which was classified as pathogenic according to the ACMG guidelines (PVS1, PM2, PP3, and PP5) [13]. This variant has previously been reported in the medical literature [9,14]. Two individuals from family 5 were compound heterozygous for the *TMC1* (NM_138691.3) variants c.1534C>T, p.(Arg512*) and c.968A>G, p.(Tyr323Cys) (hg38: chr9:72788422A>G, rs746724027). The latter is classified as likely pathogenic, according to the ACMG guidelines (PM2, PM3, PP1, and PP3), and, to the authors’ knowledge, this variant has not yet been reported in the literature. Parental testing revealed that the variants were in different alleles. Two affected individuals from families 6 and 7 were compound heterozygous for the *TMC1* (NM_138691.3) variants c.1534C>T, p.(Arg512*) and c.1763+3A>G, p.(?) (hg38: chr9:72816213A>G, rs370898981). The latter is classified as pathogenic according to the ACMG guidelines (PS3, PM3, and PM2). *TMC1* c.1763+3A>G has previously been reported in the medical literature [15,16]. Parental testing revealed that the variants were in different alleles.

Two individuals from family 8 were heterozygous for the *TMC1* (NM_138691.3) c.1714G>A, p.(Asp572Asn), (hg38: chr9:72816161G>A, rs121908072). This heterozygous *TMC1* variant and severe hearing loss segregated dominantly in this family. *TMC1* c.1714G>A, p.(Asp572Asn) is classified as pathogenic according to the ACMG guidelines (PP5, PM1, PM2, and PM5).

## 4. Discussion

In this study, we reported nine individuals from seven Finnish families with congenital ARSNHL and biallelic TMC1 variants. We also reported two individuals from one Finnish family with autosomal dominant SNHL (ADSNHL) and a heterozygous TMC1 variant. All the affected individuals with recessively inherited TMC1 variants displayed severe-to-profound congenital hearing loss associated with down-sloping audiograms, where higher frequencies are the most severely affected. This is consistent with previous publications demonstrating that pathogenic recessive TMC1 variants cause moderate-to-profound hearing loss [17,18,19,20]. The individuals affected with ADSNHL displayed progressive postlingual sensorineural hearing loss that led to profound deafness and the need for CIs. In the previous publications, ADSNHL cases showed postlingual-onset progressive hearing loss, with predominant deterioration in the higher frequencies [18,19,20,21]. Only 1 of the 11 affected individuals in the present study had balance problems. This finding is in line with previous studies that found no vestibular symptoms associated with TMC1-associated HL cases [18,19].

Previous work has shown variable auditory outcome in patients with pathogenic biallelic *TMC1* variants [19,21,22,23]. Most reports are small case series with heterogeneous samples, which makes it difficult to assess the full effect of a particular genotype. Recent study reported three patients with pathogenic compound heterozygous *TMC1* variants demonstrating excellent functional outcome after Cis [22]. Interestingly, one of the patients in this study was compound heterozygous for the Finnish enriched *TMC1* c.1534C>T, p.(Arg512*) variant [22]. Another study reported 19 patients from 12 consanguineous Saudi families with pathogenic homozygous *TMC1* variants with variable auditory outcome after Cis [19]. In the latter study, both excellent and poor results were reported for individuals with the same variants emphasizing that a significant proportion of the variance in outcomes can be due to social and environmental factors, as well as other known explanatory factors such as the duration of the deafness before the CI operation.

In our study population, the individuals who had access to CIs in early childhood, and received timely hearing rehabilitation with hearing aids and CIs, have reached a good speech perception in quiet and in noise. No age-specific reference values have been established for children for the FINSIMAT, but the two youngest individuals in the present study had SRTs in a range similar to that of typically performing early implanted CI users of their age (Willberg, personal communication). The older, early implanted CI users also reached good speech perception. The average SRT for a bilateral CI user with the FMST was −5.4 dB SNR in a large, unselected group of Finnish CI users [24]. Two of the early implanted individuals in the present study reached an even better SRT, and another individual had a SRT of −3.4 dB, which can still be considered an adequate CI rehabilitation result. Similar to the outcomes from Gallo et al. [22], our outcomes are comparable to the outcomes of individuals with *GJB2*-associated DFNB1 [25]. Both individuals with ADSNHL had poorer speech perception in noise than the early implanted individuals with ARSNHL. For the younger individual, this was most likely due to insufficient amplification with hearing aids. For the older individual, the poor SRT may be a result of prolonged deafness in the high-frequency region before the CI rehabilitation.

Even though CIs have been highly successful in restoring hearing in severe and profound hearing loss, the rehabilitation results remain highly variable with a significant proportion of the variance still unexplained [26,27]. Implementation of large gene panels and exome sequencing in clinical practice has allowed frequent detection of causative pathogenic variants in patients with hearing loss. Comparing this etiological data systematically with the outcome of CI rehabilitation, especially with speech perception measures in noise, as they are sensitive to minor differences in functional hearing, we can increase our understanding of the extent to which the underlying pathogenic gene variants explain the differences in the CI rehabilitation outcomes.

The *TMC1* c.1534C>T, p.(Arg512*) is enriched in the Finnish population, with a minor allele frequency (MAF) 24 times higher in Finns (0.003344) than in non-Finnish Europeans (0.0001395) (gnomAD v.2.1.1, https://gnomad.broadinstitute.org/, accessed on 20 February 2022) [28]. The expected number of *TMC1* c.1534C>T, p.(Arg512*) homozygote patients in Finland is (0.003344)^2^ × 5.5 million or approximately 62, and the carrier frequency of this pathogenic variant is approximately 1/300 in the Finnish population. In this cohort, the *TMC1* c.1534C>T, p.(Arg512*) variant was identified in 14 of the 18 alleles (78%) of the ARSNHL patients, and was in either a homozygous or a compound heterozygous state in all patients. Based on the gnomAD and clinical data, we suggest that the *TMC1* p.(Arg512*) variant is likely a Finnish founder variant. As hearing loss is genetically highly heterogeneous, additional similar variants will possibly be identified as enriched in the Finnish population.

The *TMC1* c.1534C>T, p.(Arg512*) causes a premature stop codon. It is predicted to cause a loss of normal protein function either through protein truncation (512 out of 760 aa) or nonsense-mediated mRNA decay. *TMC1* c.1763+3A>G, p.(?) is predicted to cause a splice site defect leading also to a loss-of-function of normal TMC1. *TMC1* c.968A>G, p.(Tyr323Cys) was identified in two affected individuals in a compound heterozygote state with a known pathogenic *TMC1* c.1534C>T, p.(Arg512*). The mother of these two affected individuals was a healthy heterozygous carrier of this *TMC1* c.968A>G, p.(Tyr323Cys) variant suggesting that *TMC1* c.968A>G, p.(Tyr323Cys) is a recessive variant leading to a functional null allele.

In contrast, *TMC1* c.1714G>A, p.(Asp572Asn) is a well-known dominantly acting *TMC1* variant [9,20,29,30]. Amino acid 572 in *TMC1* is a critical functional residue for pathogenic dominant variants [9,20,29,30]. Nucleotide 1714 of *TMC1* is located within the hypermutable CpG context, thereby explaining its location as a mutational hot spot and its occurrence in different populations [9,29,30]. Dominant variants are likely to confer a specific dominant negative or gain-of-function effect, leading to dominant inheritance [20], whereas pathogenic recessive *TMC1* variants create a functional null allele and lead to recessive inheritance through a loss-of-function mechanism. It has been proposed that two mechanisms link pathogenic *TMC1* channel variants and deafness: decreased Ca^2+^ permeability to all pathogenic variants, and decreased resting open probability in low Ca^2+^ confined to pathogenic dominant variants [31].

## 5. Conclusions

We identified *TMC1* variants c.1534C>T, p.(Arg512*), c.968A>G, p.(Tyr323Cys), and c.1763+3A>G, p.(?) with profound congenital ARSNHL in seven Finnish families, and *TMC1* c.1714G>A, p.(Asp572Asn) with postlingual progressive ADSNHL in one Finnish family. To the authors’ knowledge, the *TMC1* c.968A>G, p.(Tyr323Cys) variant is a novel variant expanding the knowledge of the genotypic spectrum of pathogenic *TMC1* variants. We also showed that the *TMC1* c.1534C>T, p.(Arg512*) variant is the most prevalent variant enriched in the Finnish population, suggesting a founder effect. To the authors’ knowledge, these are the first reported cases of *TMC1*-related SNHL in Finland.

Our study demonstrates that the individuals who had access to CIs in early childhood, and received timely hearing rehabilitation with hearing aids and CIs, have reached a good speech perception in quiet and in noise. Comparison of the genetic data with the outcome of CI rehabilitation increases our understanding of the extent to which underlying pathogenic gene variants explain the differences in CI rehabilitation outcomes. It is even possible that in the future patients can be stratified based on the underlying gene defect into categories that can predict their rehabilitation outcomes.

## Figures and Tables

**Table 1:** Clinical characteristics of patients. Patients 1–9 had recessively inherited sensorineural hearing loss associated with biallelic pathogenic recessive *TMC1* variants. Patients 10 and 11 had a dominantly inherited hearing loss associated with a heterozygous pathogenic *TMC1* variant.

	Patient 1	Patient 2	Patient 3	Patient 4	Patient 5	Patient 6	Patient 7	Patient 8	Patient 9	Patient 10	Patient 11
Family	1	2	3	3	4	5	5	6	7	8	8
Current age	21	5	42	46	53	23	18	7	0	57	21
Sex	F	F	M	M	F	M	F	M	F	F	F
Genotype	c.1534C>T, p.(Arg512*), homozygous	c.1534C>T, p.(Arg512*), homozygous	c.1534C>T, p.(Arg512*), homozygous	c.1534C>T, p.(Arg512*), homozygous	c.1534C>T, p.(Arg512*), homozygous	c.1534C>T, p.(Arg512*) and c.968A>G, p.(Tyr323Cys)	c.1534C>T, p.(Arg512*) and c.968A>G, p.(Tyr323Cys)	c.1534C>T, p.(Arg512*) and c.1763+3A>G	c.1534C>T, p.(Arg512*) and c.1763+3A>G	c.1714G>A, p.(Asp572Asn) heterozygous	c.1714G>A, p.(Asp572Asn) heterozygous
Newborn screening	Abnormal	Abnormal	NA	NA	NA	NA	Abnormal	Abnormal	Abnormal	NA	NA
OAE	No response	No response	NA	NA	NA	No response	No response	No response	No response	NA	NA
TEOAE	NA	No response	NA	NA	NA	NA	NA	NA	No response	NA	NA
AABR	NA	No response	NA	NA	NA	NA	NA	NA	No response	NA	NA
ABR (dB)	No response	l.dx: no response/l.sin: 90 (4 kHz) and click 85	NA	NA	NA	No response	No response	No response	Cochlear microphonics at 85, no neural responses at 85	NA	NA
Speech-in-noise test	SRS +10 dB SNR l.a. 99%; SRT l.dx. −8.0 dB SNR, SRT l.sin −8.6 dB SNR	SRT l.dx −5,9 dB SNR, SRT l.sin −4.9 dB SNR, SRT l.a. −6.6 dB SNR	NA	NA	NA	SRS +10 dB SNR l.a. 75%, SRT l.dx −2.3 dB SNR, SRT l.a. −3.4 dB SNR	SRS +10 dB SNR l.a. 100%, SRT l.dx −7.6 dB SNR, SRT l.a. −6.6/−7.6 dB SNR	SRT l.dx −6.3 dB SNR, SRT l.sin −4.9 dB SNR, SRT l.a. −6.2 dB SNR,	NA	SRS +10 dB SNR l.a. 94%; SRT l.sin. −1.3 dB SNR, SRT l.dx. −2.0 dB SNR, SRT l.a. −2.8 dB SNR	SRS +10 dB SNR l.a. 88%, SRT l.a. +0.4 dB SNR
Age at diagnosis	7 m	2 m	From birth	1 y 8 m	From birth	From birth	1 m	8 m	From birth	7 y	7 y
Severity	Profound	Profound	Profound	Profound	Profound	Profound	Profound	Profound	Profound	Profound	Severe
Progression	No	No	No	No	No	No	No	No	No	Yes	Yes
Hearing aids (from the age)	Yes (7 m)	Yes (4 m)	No	No	Yes (1.5 y)	Yes (1 y 4 m)	Yes (3 m)	Yes (9 m)	Yes (4 m)	No	Yes
Cochlear implant (from the age)	Yes (1 y 5 m) (right) and 13 y (left)	Yes (1 y)	No	No	No	Yes (2 y 1 m (right) and 20 y 9 m (left)	Yes (1 y (right) and 15 y 5 m (left)	Yes (1 y 5 m (right) and 1y6m (left)	Planned (8–9 m)	Yes (41 y (left) and 51 y (right)	No
Balance problems	No	No	No	No	No	Yes	No	No	NA	No	No
Language perception	Good progress with CI use	Good progress with CI use	Sign language	Sign language	Sign language	Good progress with CI use	Good progress with CI use	Good progress with CI use	NA	Normal spoken language	Normal spoken language

Abbreviations: OAE, otoacoustic emission; TEOAE, transient evoked OAE; ABR, auditory brainstem response; AABR, automated ABR; SRS, speech recognition score; SNR, signal-to-noise ratio; SRT, speech reception threshold; CI, cochlear implant; l.a., both sides; l.dx., right side; l.sin., left side.

## Data Availability

The reported variants were submitted to the LOVD database hosted at Leiden University Medical Center, The Netherlands.
